# Is Emergency Appendicectomy Better Than Elective Appendicectomy for the Treatment of Appendiceal Phlegmon?: A Review

**DOI:** 10.7759/cureus.12045

**Published:** 2020-12-12

**Authors:** Asma Ahmed, Shah Huzaifa Feroz, Jerry Lorren Dominic, Abilash Muralidharan, Pragatheeshwar Thirunavukarasu

**Affiliations:** 1 General Surgery, Ramaiah Medical College and Hospital, Bangalore, IND; 2 General Surgery, Jawaharlal Nehru Medical College, Aligarh, IND; 3 General Surgery, Larkin Community Hospital, Miami, USA; 4 General Surgery, South Texas Health System, Edinburg, USA; 5 Internal Medicine, Kiruba Hospital, Coimbatore, IND; 6 Surgical Oncology, Cancer Treatment Centers of America, Boca Raton, USA

**Keywords:** appendix, appendiceal phlegmon, appendiceal mass, emergency appendicectomy, elective appendicectomy, laparoscopic appendicectomy

## Abstract

Appendiceal phlegmon is considered to be sequelae to acute appendicitis which presents as an appendiceal mass composed of the inflamed appendix, the adjacent bowel loops, and the greater omentum. The definitive diagnosis can be obtained by a CT scan of the abdomen. Though conservative management was the most practiced approach, recent studies have shifted the trends towards immediate appendicectomy for the management of appendiceal phlegmon. Thus, the management of appendiceal phlegmon has been debatable. Evidence to support this review was gathered via the PubMed database as this database uses the Medline, PubMed Central, and NLM databases and also offers a quick diverse search with up-to-date citations and numerous open-access free articles focused on Medicine. We did not include other databases like Google Scholar, Embase, and Scopus due to its limited access to free articles, recent articles, and citation information. Search terms used were combinations of "Appendicitis," "Appendiceal phlegmon", "Appendiceal phlegmon (AND) appendicectomy ". The resultant studies were reviewed and cross‐referenced for additional reports. Emergency appendicectomy is defined as appendicectomy carried out during the same, initial admission. An elective or interval appendicectomy is an appendicectomy carried out four to six weeks after the initial episode at a later admission. The interval is bridged by antibiotics and conservative management. Emergency appendicectomy is considered to have a higher rate of complications when compared to conservative management for appendiceal phlegmon. However, interval appendicectomy requires multiple re-admissions, leads to delayed diagnosis of any underlying pathology, and an increased risk of recurrent appendicitis. In our review, we aimed to compare and contrast the effectiveness of the different treatment modalities available for appendiceal phlegmon. Though the meta-analyses showed an increased association of complications with emergency appendicectomy, they included studies conducted before the laparoscopic era. Emergency appendicectomy decreases the financial burden, re-admission rate, and aids in the early diagnosis of any underlying pathology. In the laparoscopic era, we can consider the shifting trends towards emergency appendicectomy for the management of appendiceal phlegmon.

## Introduction and background

“The indications for surgical interference in appendicitis are not always clear. But the abdomen is much more frequently left untouched than it should be, and that an operation is too often deferred until practically useless” - Sir William Osler

Appendicitis is one of the most common surgical emergencies with a prevalence encountered in the emergency room. It occurs most commonly in the 2nd and 3rd decades of life and carries a higher risk in men as compared to women [1]. The typical symptoms are migration of pain from the periumbilical region to the right iliac fossa associated with nausea, vomiting, anorexia, fever, and usually leukocytosis.

Appendicitis can present itself in two ways and the most common presentation with a prevalence of 20%-30% is the acute inflammation of the appendix which can eventually rupture and cause localized or generalized peritonitis. The second and less common type is the appendiceal phlegmon. It occurs in around 10% of the cases and has a much slower course. When an inflamed appendix perforates, the infection is localized by the omentum and bowel loops which present as an appendiceal mass or phlegmon [2].

The widely accepted treatment for acute appendicitis is an emergency appendicectomy but the management of an appendiceal phlegmon is still controversial. Traditionally, the phlegmon is managed conservatively with antibiotics followed by interval appendectomy four to six weeks later. The initial conservative management is recommended as appendicectomy in the background of acute inflammation can be time-consuming and can lead to bowel damage and fistula formation. The other treatment modality is emergency appendicectomy as it averts the need for readmission and provides an early cure. It also helps in earlier identification of any underlying pathology like malignancy or colitis, if present [2].

In this study, we aim to review and assess the effects in terms of morbidity and mortality of the two treatment modalities for appendiceal phlegmon - emergency and elective appendectomy.

## Review

Methods

Evidence to support this review was collected from the PubMed database. The search terms used were “Appendicitis”, “Appendiceal Phlegmon” and “Appendiceal phlegmon (AND) emergency appendicectomy and "Appendiceal Phlegmon(AND) (elective appendicectomy (OR) delayed appendicectomy (OR) interval appendicectomy)” with a filter of the 20-year publication date and human studies (Table 1). Studies discussing and reviewing the management of appendiceal phlegmon by emergency appendicectomy and by elective appendicectomy/delayed appendicectomy/interval appendicectomy were considered. All the data were collected after a thorough review of the articles.

**Table 1:** Number of articles found on the PubMed Database related to our keywords.

Keyword	Database	Number of Articles
Appendicitis	PubMed	11,900
Appendiceal Phlegmon	PubMed	94
Appendiceal Phlegmon (AND) emergency appendicectomy	PubMed	18
Appendiceal Phlegmon (AND) (elective appendicectomy (OR) delayed appendicectomy (OR) interval appendicectomy)	PubMed	19

Discussion

Acute appendicitis is the inflammation of the appendix which is caused by the obstruction of the appendiceal lumen by faecolith, foreign body, lymphoid hyperplasia, or malignancy. This causes increased intraluminal pressure eventually leading to appendiceal ischemia and necrosis [1].

Complicated appendicitis includes appendiceal phlegmon and localized appendiceal abscess. An appendiceal phlegmon presents as a palpable appendiceal mass which is composed of the inflamed appendix, its adjacent viscera, and the surrounding greater omentum [3]. The diagnosis of phlegmon is suspected in patients with a palpable mass or with symptoms lasting more than three days and is more common in children, especially in those aged less than five years [3]. It is often undiagnosed preoperatively and the proportion of all patients with appendicitis treated for appendiceal phlegmon is 3.8%-5.0%. The risk of perforation is negligible within the first 12 hours of untreated symptoms but then increases to 8.0% within the first 24 hours [4-9].

Appendiceal phlegmon occurs in 2%-10% of cases of appendicitis. It has greater morbidity in children and the elderly as these sub-groups have a delayed presentation of the appendiceal mass [10]. The definitive diagnosis can be obtained by a combination of the patient’s history, clinical examination, laboratory investigations, and mainly by radiological investigations like abdominal ultrasound or CT scan of the abdomen. Appendiceal phlegmon has low mortality of less than 1% but has higher morbidity than acute appendicitis^ ^[10].

The most commonly used imaging modalities for the diagnosis of appendicitis are CT scans and Ultrasound of the abdomen. Recent meta-analyses have concluded that CT scan is superior to ultrasound in the diagnosis of appendicitis and specifically in the diagnosis of appendicular mass or abscess [11-14]. The normal length of the appendix is 8-10 cm and >6 mm dilatation of the appendiceal diameter is diagnostic of acute appendicitis. Phlegmon, abscess, extraluminal air, and appendiceal wall enhancement defect are more indicative of perforated appendicitis [15-17].

The two different treatments available for complicated appendicitis can be early or delayed appendicectomy. Early appendicectomy can be defined as any appendicectomy that is performed immediately or a few days later within the same hospitalization. Delayed appendicectomy or interval appendicectomy can be defined as initial conservative management followed by appendicectomy four to six weeks later at a different admission [10]. The conservative treatment also known as the Oschner Sherren Regimen includes hospitalization, antibiotics, analgesics, recording the daily size of the mass, and strict monitoring of the vitals and general state of the patient. Common complications associated with surgery are intra-abdominal abscesses, incisional hernias, wound infections, and bowel obstructions [10].

The optimal approach to complicated AA with phlegmon is debatable between emergency appendicectomy and elective appendicectomy.

Emergency Appendicectomy

Traditionally, the management of appendiceal phlegmon is initial conservative management with antibiotics followed by interval appendectomy. However, recent studies have questioned this conventional approach and advocated emergency appendicectomy for appendiceal phlegmon.

A prospective study conducted by Bahram showed that out of 34 patients who presented with an appendiceal phlegmon, emergency appendicectomy outweighed the results of an interval appendicectomy. The duration of hospital stay ranged from two to four days with the mean hospital stay was 3 ± 0.25 days. The postoperative complications were seen in 12 out of 34 patients [18]. In a study by Samuel et. al., 34 patients with an appendicular mass underwent emergency appendicectomy and had an identifiable appendix at the time of surgery. The mean length of hospital stay was 4.8 ± 0.4 days. Three patients who developed postoperative infections were treated with oral antibiotics and one patient required drainage^ ^[19].

These findings are consistent with a study by Senapathi et al., which showed that there were no significant differences between the two groups who underwent emergency appendicectomy versus those who underwent interval appendicectomy based on the post-operative stay, hospital stay, and postoperative infections [20].

Young et al., in a retrospective study, demonstrated that patients managed with emergency appendicectomy had a significantly lower incidence of major bowel resection for non-malignant pathology (3.3% vs. 17.1%, p< 0.048), and two patients who underwent bowel resection required an ileostomy. No significant difference was found in length of hospital stay (p = 0.24), though the patients undergoing emergency appendicectomy had a significantly longer post-operative hospitalization (4.3 [2.2, 6.8] vs. 1.9 [0.3, 5.9], p< 0.03) [21].

In a study by Deeldar et al., we also found an acceptable incidence of, mostly minor, complications (17.6 %) after immediate surgery. In the operative group, while the rate of extensive (ileocecal+ hemicolonic) resection was 30.8 %, there were no recurrences, and the average length of stay was relatively short (9.2 days); few (5.9 %) patients had to be re-admitted, and a histopathological diagnosis was established in most (91.2 %) patients [2].

A meta-analysis conducted by Gavrilidis et al. showed that the hospital stay was shortened by one day with the emergency appendicectomy approach when compared to the conservative management for appendiceal phlegmon.^ ^However, the incidence of overall complications, abdominal/pelvic abscesses, and wound infections were higher in the patients who underwent emergency appendicectomy as compared to those who underwent conservative management with a p-value of 0.0002, 0.02 and <0.001, respectively [22]. A case report by Elkbuli et al. showed how a 19-year-old patient managed conservatively required recurrent hospitalizations and increased the burden on healthcare [1]. In a randomized control trial by Blakely et.al, involving children, comparing early appendectomy with interval appendectomy, it was found that those treated with early appendectomy returned to normal activities an average of five days earlier (p < 0.001) [23].

Elective or Interval Appendicectomy

In a retrospective study by Aranda Narvaez et al., the stratification of the infectious risk according to the National Nosocomial Infections Surveillance System (NNIS) index showed a greater percentage of patients with a high-risk NNIS (≥ 2) in the group that underwent emergency appendicectomy as compared with the group with conservative management for appendiceal phlegmon (80 vs. 28.6%, p < 0.03). The study showed no significant differences between the groups with respect to operative time (p < 0.91) [24].

In a meta-analysis conducted by Simillis et al., the results demonstrated that the patients who underwent conservative management had lesser complications like wound infections, abdominal/pelvic abscesses, and bowel obstructions in comparison to the group that underwent emergency appendicectomy [25].The analysis reported on complications higher in the emergency appendicectomy group compared with the conservative management group (OR, 0.24; 95% CI, 0.13-0.44; p < 0.001), and heterogeneity between studies was observed (p < 0.001). The incidence of bowel obstruction was found to be higher in the emergency appendicectomy group (OR, 0.35; 95% CI, 0.17-0.71; p = 0.004), with no heterogeneity between studies. The risk of reoperations was higher in the group with emergency appendicectomy (OR, 0.17; 95% CI, 0.04-0.75; p = 0.02) [25].

The meta-analysis by Andersson et al. showed that immediate appendicectomy was associated with thrice the morbidity of conservative treatment. Conservative management was successful in about 93% of the patients [26]. The risk of recurrence after nonsurgical treatment was less than 10% and was due to an appendicolith. The risk of recurrence was 7.2% (CI: 3.2-11.0) in cases of conservative management which was lower than 7.4% (CI: 3.7-11.0) in the analysis of studies that underwent emergency appendicectomy. The recurrence is characterized by a milder course than the primary attack in most cases [27].

Despite numerous studies on the preferred management of the appendiceal phlegmon, the most optimal approach is still debated.

Conservative management followed by interval appendicectomy had been the mainstay for the treatment of appendiceal phlegmon. It has been found to be successful in at least 90% of the patients [25]. Emergency appendicectomy could cause injury to the bowel in the setting of an inflammatory process whereas conservative management helps to localize the inflammatory process. This theory advocated reducing the number of surgical complications. Nonsurgical management reduces the morbidity of the patient by decreasing the length of hospital stay and avoids surgical complications requiring ileal or bowel resection. However, nonoperative management has its own disadvantages. It requires rehospitalization for an interval appendicectomy which increased the financial burden on healthcare. The interval between the first admission and the time of delayed appendicectomy can be marked by recurrent attacks of appendicitis. One of the main drawbacks of this procedure is that delayed treatment can cause a delayed diagnosis of any underlying condition like Crohn’s disease or a malignancy which is seen in around 2% of patients [27].

With the advent of new surgical techniques, there is a paradigm shift towards emergency appendicectomy for the treatment of appendiceal phlegmon. This modality of treatment requires only a single admission and without the risk of recurrent appendicitis. It reduces the morbidity of rehospitalization and improves the quality of life in a patient. It also aids in the early diagnosis of underlying conditions like malignancy which can warrant an ileal resection or right-sided hemicolectomy. However, the surgery itself can be challenging, in the identification of the appendix, avoiding injury to the bowel, suturing the stump in the background of edematous and friable tissues. This has been one of the main drawbacks of this treatment but with newer developments in surgical techniques like laparoscopic and robotics surgery, emergency appendicectomy has become a much safer and feasible option for appendiceal phlegmon (Table 2).

**Table 2:** Comparison between emergency appendicectomy and elective appendicectomy.

EMERGENCY APPENDICECTOMY		ELECTIVE APPENDICECTOMY	
Advantages	Disadvantages	Advantages	Disadvantages
Reduces length of hospital stay in the initial admission [19]	Operative difficulty [21]	Safer with a lower risk of iatrogenic bowel injury [24]	Failure of initial conservative management [18]
Reduces financial burden [10]	Increased risk of morbidity [27]	Recurrent attacks are milder than the primary attack [27]	Higher incidence of recurrent attacks of appendicitis [18,20]
Comparatively lesser intraoperative adhesions [19]	Higher risk of surgical site infections [24]	Easier delineation of the intra-operative anatomy [25]	Missed diagnosis of an underlying condition like colitis or malignancy [2,20]
Lesser rate of re-admissions [1,2]	Increased risk in organ-space infections [24]	The shorter length of hospital stay during both the admissions [24]	Failure of this management can lead to surgical admission requiring bowel resection [21]
Better health-related quality of life [10]	Higher risk of occurrence of abdominal/pelvic abscess [25]		Loss of patients to follow-up [2]
Laparoscopic approach can reduce the risk of post-operative complications [20]			The increased financial burden due to multiple admissions [1]

Limitations

We were able to review articles and conclude about the efficiency of emergency appendicectomy over delayed appendicectomy in the management of appendiceal phlegmon. We included only free articles available on the PubMed database and did not include articles from other databases like Google Scholar, EMBASE, and Scopus. The quality of studies was not gauged prior to their inclusion in the article.

## Conclusions

The review of studies comparing the efficacy of different modalities of treatment for appendiceal phlegmon is highly varied. There have been advantages and disadvantages of both emergency and elective appendicectomy mentioned in the studies. The meta-analysis conducted by Similis et al. and Andersson et al. show that nonoperative management is preferred as compared to emergency appendicectomy. However, these meta-analyses are based on studies that have mostly been conducted before the laparoscopic era. Furthermore, the studies conducted with laparoscopic emergency appendicectomy in consideration for appendiceal phlegmon show positive results but are limited by the small subset of patients considered. In a well-equipped healthcare setting, an emergency appendicectomy can be considered a quick, safe, feasible, and cost-effective treatment for the management of appendiceal phlegmon. Though we can see a paradigm shift for the management of appendiceal phlegmon from delayed appendicectomy to emergency appendicectomy in the laparoscopic era, further qualitative studies are required to substantiate this model of management.
